# Case report: Inhaled nitric oxide rescued a hypoxemia patient caused by dermatomyositis complicated with interstitial pneumonia

**DOI:** 10.3389/fmed.2024.1371183

**Published:** 2024-05-03

**Authors:** Xiaoyan Wu, Ruiqiang Zheng, Zhanqi Zhao

**Affiliations:** ^1^Medical College of Yangzhou University, Department of Critical Care Medicine, Northern Jiangsu People’s Hospital, Yangzhou, China; ^2^School of Biomedical Engineering, Guangzhou Medical University, Guangzhou, China; ^3^Department of Critical Care Medicine, Peking Union Medical College Hospital, Beijing, China; ^4^Institute of Technical Medicine, Furtwangen University, Villingen-Schwenningen, Germany

**Keywords:** dermatomyositis, interstitial pneumonia, inhaled nitric oxide, hypoxemia, ventilation/perfusion matching

## Abstract

Interstitial pneumonia is the most common and serious secondary lesion of dermatomyositis. In some cases, patients may develop severe acute pneumonia that can quickly progress to respiratory failure, resulting in high mortality rates. A 57-year-old woman with dermatomyositis and interstitial pulmonary fibrosis experienced severe hypoxemia due to pulmonary infection. Despite receiving various treatments after entering the intensive care unit (ICU), such as anti-infection therapy, lung recruitment, prone position ventilation, sedative and muscle relaxation, the patient’s oxygen saturation continued to decline. Electrical impedance tomography (EIT) monitoring revealed that prone position could not improve ventilation homogeneity. However, the patient’s ventilation/perfusion (V/Q) matching significantly improved 10 min after initiation of supine position ventilation combined with inhalation of nitric oxide (iNO). The patient’s PaO_2_/FiO_2_ (P/F) ratio increased from 86 mmHg to 150 mmHg at 30 min post-treatment. iNO treatment continued for 2 days. Then the patient’s condition improved and she was successfully weaned off the ventilator with rigorous monitoring and symptomatic care. The implementation of mechanical ventilation combined with iNO therapy rapidly improved V/Q matching and oxygenation in a patient with hypoxemia caused by dermatomyositis complicated with interstitial pneumonia. This approach successfully avoided the need for invasive extracorporeal membrane oxygenation (ECMO) support.

## 1 Introduction

Interstitial pneumonia is a common complication of inflammatory myopathy, particularly prevalent in patients with dermatomyositis, with an incidence rate of up to 65%. However, the incidence of the disease in China is still unknown and it is a rare disease. Interstitial pneumonia can result in respiratory failure, pulmonary hypertension (PH), and secondary pulmonary infections, making it the leading cause of death in dermatomyositis patients, with a fatality rate of up to 40% (1).

Inhaled nitric oxide (iNO) is a selective pulmonary vasodilator, which can improve the ventilation/perfusion (V/Q) matching in patients with acute respiratory failure, potentially improving oxygen and reducing pulmonary vascular resistance (2). iNO is used extensively worldwide as a rescue agent in acute hypoxaemic respiratory failure (AHRF) and acute respiratory distress syndrome (ARDS) patients, although many clinical studies have shown that it cannot improve mortality (3, 4). Recent studies have shown that iNO has the potential to improve oxygenation by optimizing V/Q matching and ameliorating resting and/or exercise-induced PH in patients with fibrotic interstitial lung disease (5, 6). Here we first reported a case of effective iNO use in a hypoxemia patient caused by dermatomyositis complicated with interstitial pneumonia.

## 2 Case report

**Figure 1:**
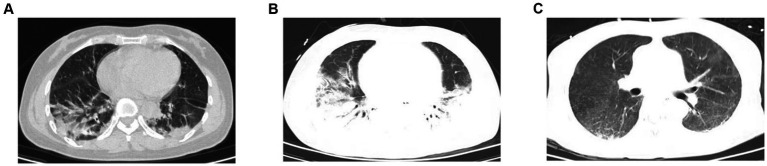
Chest CT of the patient. **(A)** Chest CT showed increased texture in both lungs with patchy/streaky hyperdense shadows with blurred edges (before hospitalization). **(B)** Chest CT exhibited bilateral pneumonia with local consolidation (before iNO). **(C)** Chest CT re-examination displayed a significant improvement in the absorption of inflammation compared to earlier (on day 13).

A 57-year-old woman with dermatomyositis complicated with pulmonary interstitial fibrosis was admitted to the Emergency Medicine Department due to pulmonary infection 6 months ago. The patient had a history of dermatomyositis, more than 8 months ago, she had experienced paroxysmal wheezing, chest tightness, asthma, intermittent coughing, and expectoration of white phlegm without clear causes. The local hospital tested positive for antinuclear antibodies and RO52. Pulmonary function examinations in the outpatient setting revealed severe mixed ventilation dysfunction and a negative bronchodilator test. Chest computed tomography (CT) scans revealed bilateral pneumonia (Figure 1A). Then she was treated thrice daily with two tablets of hydroxychloroquine and three tablets of acetic acid prednisolone. However, the treatment was discontinued after irregular administration. Almost 6 months ago, these symptoms worsened. The patient visited the emergency ward of our hospital and was subsequently admitted to the Emergency Medicine Department. After admission, she received high-flow oxygen inhalation, empirical anti-infection treatment with piperacillin and tazobactam, methylprednisolone, and therapy to relieve asthma and expectoration. Despite 10 days of treatment, her condition did not improve. Chest CT scan revealed bilateral pneumonia with localized consolidation (Figure 1B). The maintenance of oxygen saturation was challenging, with decreased blood pressure and aggravated respiratory and circulatory failure. After undergoing tracheal intubation and ventilator-assisted ventilation, she was transferred to intensive care unit (ICU). On admission, the arterial oxygen partial pressure was 87.2 mmHg, the arterial carbon dioxide partial pressure was 45.3 mmHg, pH was 7.293, lactic acid was 1.6 mmol/L, and the PaO_2_/FiO_2_ (P/F) ratio was 145.4 mmHg. A chest X-ray revealed multiple exudates in both lungs and pleural effusion on both sides. Echocardiography indicated mild reflux of the aortic valve, tricuspid valve, and mitral valve, with a pulmonary artery systolic pressure of 35 mmHg.

The patient received intensive care in ICU, and tracheal intubation was connected to a ventilator for assisted ventilation in synchronized intermittent mandatory ventilation + pressure-support ventilation mode (SIMV+PSV mode): positive end-expiratory pressure 12 cm H_2_O, tidal volume 360 mL (6.3 mL/kg), respiratory rate (RR) 24 times/min, fraction of inspired oxygen 60%, and pressure support (PS) 15 cm H_2_O. Sputum culture and bronchoalveolar lavage fluid next-generation sequencing revealed the presence of *Pseudomonas aeruginosa*, *Enterococcus faecalis*, and *Haemophilus parainfluenzae*, along with fungi including *Candida carinii* and *Candida glabrata*. Consequently, targeted anti-infection treatment was initiated using imipenem, linezolid, and caspofungin. Anti-nuclear antibodies were detected at 1:80, and the anti-Ro52 antibody tested positive. The diagnosis indicated an idiopathic inflammatory myopathy spectrum with positive anti-EJ antibody IgG and anti-RO-52 antibody IgG. Treatment involved high-dose immunoglobulin (20 g × 3 days) and high-dose methylprednisolone (500 mg × 3 days), followed by a shift to 80 mg QD × 10 days. Currently, the patient is maintaining a regimen of methylprednisolone 60 mg QD, tacrolimus 1 mg Q12h, pirfenidone for anti-pulmonary interstitial fibrosis. Norepinephrine was administered for circulation maintenance, and continuous renal replacement therapy (CRRT) was administered for acute kidney injury, anuria, and acidosis. Monitoring the patient’s respiratory mechanics revealed a plateau pressure of 33 cm H_2_O and a driving pressure of 19 cm H_2_O. Consequently, the tidal volume was reduced to a minimum of 4.1 mL/kg, and the respiratory rate was increased to 35 times/min to maintain effective alveolar ventilation. Despite these efforts, the oxygen saturation of the patient progressively worsened, resulting in significant respiratory distress and a P/F ratio as low as 100 mmHg. To address this, we implemented lung recruitment and prone position ventilation combined with sedative and muscle relaxation. However, after just 1 h in the prone position, the patient experienced another drop in oxygen saturation. Over the disease course, the pulmonary artery pressure (PAP) reached up to 56 mmHg. Given the severity of pulmonary lesions complicated with severe hypoxemia, carbon dioxide retention, and poor lung compliance, with platform pressure and driving pressure exceeding the safe range, extracorporeal membrane oxygenation (ECMO) treatment was recommended but refused by the family due to cost and risk.

**Figure 2:**
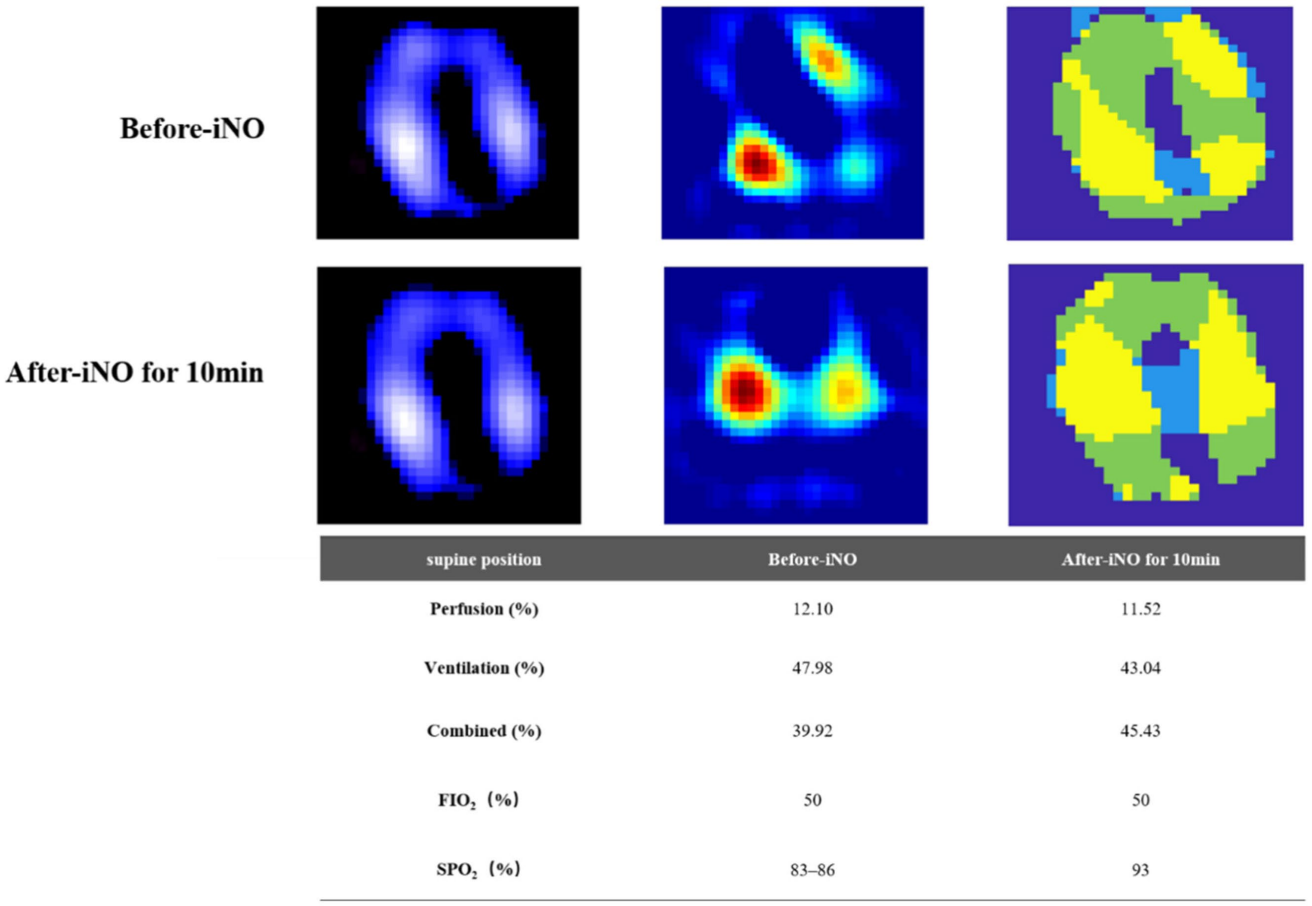
Ventilation and perfusion before and after inhaling NO in the supine position assessed by electrical impedance tomography (EIT). First row, before inhaled nitric oxide (iNO). Second row, after iNO for 10 min. First column, ventilation distribution, ventilated regions were marked in purple to white (high). Second column, perfusion distribution, perfused regions were marked in green to red (high). Third column, distribution of regional ventilation/perfusion (V/Q) matching. Regions with perfusion only were marked in blue (shunt), ventilation only were marked in green (dead space), and V/Q matching in yellow. The bottom table, changes of V/Q matching before and after iNO therapy.

**Figure 3:**
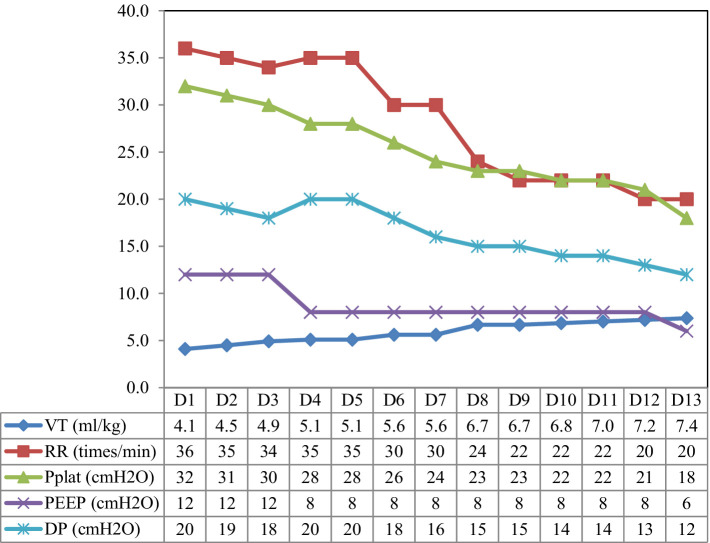
The parameter settings of the patient’s ventilator. VT, tidal volume; RR, respiration rate; Pplat, plateau pressure; PEEP, positive end-expiratory pressure; DP, driving pressure.

**Figure 4:**
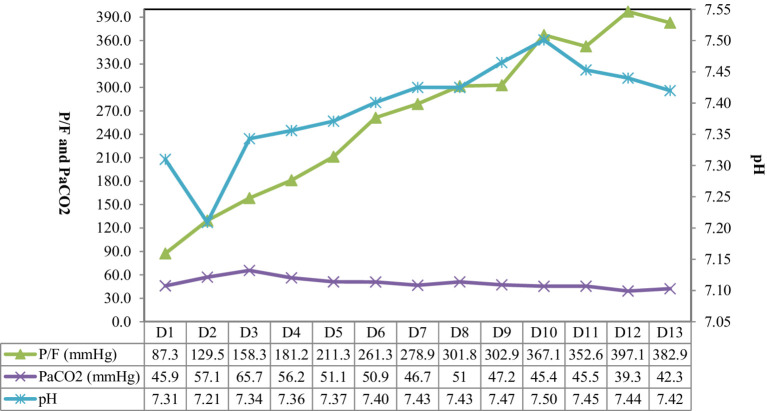
The gas exchange of the patient. P/F, PaO_2_/FiO_2_ ratio.

Electrical impedance tomography (EIT) indicated that the patient’s dorsal ventilation ratio was −8 to −5%, suggesting that prone positioning did not improve ventilation homogeneity. Consequently, the patient was returned to supine position for ventilation; however, the saturation of peripheral oxygen (SPO_2_) remained in the range of 83–86% with a P/F ratio of 86 mmHg. The patient received iNO (INOwill N200 Nitric Oxide Generator and Delivery System, Novlead Biotechnology, China) at 20 parts per million (ppm) for 30 min, resulting in a blood gas analysis demonstrating a P/F ratio of 150 mmHg and SPO_2_ of 93%. During the supine position and supine position with iNO, the EIT-monitored V/Q ratio showed a decrease in ventilation from 47.98 to 43.04% after iNO, the pulmonary shunt decreased from 12.10 to 11.52%. Ventilation and perfusion became more closely matched (from 39.92 to 45.43%) (Figure 2). iNO treatment continued at 10–20 ppm in the first two days, and no further prone position ventilation was performed. Under close monitoring, ventilator parameters were adjusted based on the respiratory mechanics of the patient and gas exchange (Figure 3). After 13 days of treatment (Table 1), there was a significant improvement in gas exchange (Figure 4) and chest CT (Figure 1C), with PAP decrease to 23 mmHg. The patient was successfully weaned from ventilation on day 14.

**Table 1:** Changes in vasopressors, sedatives, muscle relaxants, CRRT, and urine volume during the disease course.

	Baseline^a^	D1	D2	D3	D4	D5	D6	D7	D8	D9	D10	D11	D12	D13
Norepinephrine (μg/min)		3	3											
Midazolam (mg/h)		12	12	12	8	6	4	6						
Dexmedetomidine (μg/kg/h)									0.4	0.4	0.4	0.4	0.4	0.2
Remifentanil (μg/kg/min)		0.16	0.2	0.2	0.2	0.16	0.16	0.12	0.14	0.04	0.06	0.04	0.04	0.02
Bencisatracuriμm (mg/h)			5	5										
CRRT		No	Yes	Yes	Yes	Yes	Yes	Yes	Yes	Yes	Yes	No	No	No
Pulmonary artery systolic pressure (mmHg)	35	56		50			38			23				
Urine volume (mL/24 h)		750	300	130	125	100	120	85	140	85	1,010	1,620	1,030	1,340
Fluid balance (mL/24 h)		3,206	550	−1,860	−365	−425	−339	−130	−1,419	−755	−413	597	883	690

a Baseline value represented the patient’s pulmonary artery systolic pressure before admission.

## 3 Discussion

In clinical studies of adult ARDS, inhalation of NO (5–20 ppm) has been shown to dilate pulmonary arteries, improve V/Q ratio, and enhance arterial oxygen saturation in a short period, while reducing pulmonary artery pressure (7–9). Approximately 13% of patients with severe ARDS globally have received iNO treatment due to its rapid improvement in oxygen saturation (10). Hypoxia, hypercapnia, acidosis, vasopressors, endothelial dysfunction, airway collapse and excessive airway pressure during mechanical ventilation may lead to increased pulmonary vascular resistance in ARDS patients, and further lead to increased right ventricular afterload, which could be seen in as many as 25% ARDS patients (11). In the present study, the patient developed a secondary infection of basal interstitial pneumonia complicated with ARDS, which was characterized by an acute increase in pulmonary artery pressure and rapid deterioration of oxygenation. Studies on systemic vasodilators in ARDS have shown that they may have an indiscriminate vasodilatory effect on the entire pulmonary vascular system, causing the vasodilation in both ventilated and non-ventilated lung areas, and may increase the V/Q matching, potentially leading to worsening respiratory gas exchange (12–14). Conversely, iNO may play a role in the treatment of ARDS, because it determined the selective dilation of blood vessels in ventilated lung units, thus improving V/Q matching. This selective effect was due to the short half-life of NO. The improvement of the V/Q ratio was associated with better gas exchange (15, 16). In addition, iNO could also reduce pulmonary vascular resistance and right ventricular afterload by dilating pulmonary blood vessels. Yamaguchi et al. (17) reported that iNO could be used to predict the clinical efficacy of drugs for PH and guide clinical treatment. Rapid inactivation of iNO did not affect the systemic vascular system, thus preventing systemic hypotension and the subsequent risk of organ hypoperfusion and ischemia.

Prone position ventilation is a salvage treatment for moderate-to-severe ARDS, significantly reducing mortality (18, 19). However, not all patients are affected by prone the prone position. Studies have shown that prone position ventilation can improve oxygenation in 60% of ARDS patients. The patient in this study suffered from basic pulmonary fibrosis, the disease had started more than 10 days before she was admitted to our department, and had progressed to advanced ARDS. EIT monitoring found that the cause of hypoxemia in the patient was more closely related to dead space ventilation. For patients with early ARDS and mainly intrapulmonary shunt, prone position may be more effective, so it was difficult to improve the patient’s hypoxemia (20).

At present, iNO therapy is mainly used in ARDS and neonatal refractory PH, but there are few research on chronic lung diseases such as interstitial lung disease. In recent years, increasing evidence has demonstrated that NO has a variety of important biological effects, such as inhibiting the abnormal proliferation of vascular smooth muscle and fibroblasts by antagonizing certain mitogen (21), increasing pulmonary blood flow, promoting oxygenation, alleviating pulmonary edema, reducing neutrophil aggregation, inhibiting its peroxidation and activity (22), and antiviral effects (23). Wang et al. (24) reported that pre-irradiation administration of iNO in rats significantly reduced the incidence and severity of acute radiation interstitial pneumonia, decreased mortality, and delayed and attenuated pulmonary fibrosis. However, the efficacy of iNO post-irradiation was found to be diminished. Nathan et al. (6) demonstrated that iNO could improve oxygenation and moderate/vigorous physical activity in patients with interstitial pneumonia complicated with PH.

For the past few years, some clinical reports have shown that iNO therapy can significantly improve the V/Q mismatch in patients with ARDS caused by aspiration pneumonia and acute respiratory failure within one hour, which were found by EIT monitoring (25, 26). The patient in this case study developed severe ARDS, concomitant PH, and profound hypoxemia as a result of an infection superimposed on pre-existing interstitial pneumonia. Despite receiving conventional treatments such as high-condition ventilator support, lung recruitment, and prone position, her condition deteriorated rapidly. ECMO is an effective treatment for severe respiratory failure. However, the mortality rate for severe ARDS can be as high as 35–45% even with ECMO support (27). Considering the high cost and risks associated with ECMO, including bleeding and secondary infection, the patient did not undergo ECMO treatment. Instead, she received mechanical ventilation combined with iNO treatment, which gradually improved her lung condition and provided time for treating the underlying disease and controlling the infection. Throughout the treatment, methylhemoglobin levels remained at 0.3–0.5%, renal function improved gradually, and CRRT was discontinued. When facing difficulties in clinical treatment for these patients, the reintroduction of iNO has shown promising results, which may be attributed to its vasodilatory effects on improving V/Q ratio, reducing pulmonary arterial pressure, and its anti-fibrotic properties. This provides clinicians with an effective treatment approach.

## 4 Conclusion

This is the first report demonstrating the rapid improvement of V/Q matching and oxygenation after iNO treatment in a severe hypoxemia patient caused by dermatomyositis combined with interstitial pneumonia. The current case indicated the potential practical value of using iNO as a salvage measure for the treatment of refractory hypoxemia in cases of interstitial pneumonia combined with severe infection and pulmonary hypertension.

## Data availability statement

The raw data supporting the conclusions of this article will be made available by the authors, without undue reservation.

## Ethics statement

The requirement of ethical approval and the studies involving humans was waived by Ethics Committee of North Jiangsu People’s Hospital. The studies were conducted in accordance with the local legislation and institutional requirements. The participants provided their written informed consent to participate in this study. Written informed consent was obtained from the individual(s) for the publication of any potentially identifiable images or data included in this article.

## Author contributions

XW: Writing – original draft, Writing – review & editing. RZ: Writing – original draft, Writing – review & editing. ZZ: Data curation, Software, Visualization, Writing – review & editing.
